# Advances in Fiber-Based Wearable Sensors for Personal Digital Health Monitoring

**DOI:** 10.3390/ma16237428

**Published:** 2023-11-29

**Authors:** Jingge Liu, Junze Zhang, Jing Liu, Weiwei Sun, Weiqiang Li, Hongqiang Shen, Lingxiao Wang, Gang Li

**Affiliations:** 1 National Engineering Laboratory for Modern Silk, Soochow University, Suzhou 215123, China; 20235215021@stu.suda.edu.cn (J.L.); 20224015002@stu.suda.edu.cn (W.S.); 20224215034@stu.suda.edu.cn (L.W.); 2 Nanotechnology Center, Research Institute for Intelligent Wearable Systems, The Hong Kong Polytechnic University, Kowloon 999077, Hong Kong; 22068974r@connect.polyu.hk; 3 Department of Materials, University of Manchester, Manchester M13 9PL, UK; jing.liu-42@postgrad.manchester.ac.uk; 4 Nantong Textile & Silk Industrial Technology Research Institute, Nantong 226300, China

**Keywords:** wearable sensors, fiber-based sensor, personal digital health, biophysical signal, biochemical signal

## Abstract

With the continuous growth of the global economy, an increasing concern has emerged among individuals with regard to personal digital health. Smart fiber-based sensors meet people’s demands for wearable devices with the advantages of excellent skin-friendliness and breathability, enabling efficient and prompt monitoring of personal digital health signals in daily life. Furthermore, by integrating machine learning and big data analysis techniques, a closed-loop system can be established for personal digital health, covering data collection, data analysis, as well as medical diagnosis and treatment. Herein, we provide a review of the recent research progress on fiber-based wearable sensors for personal digital health. Firstly, a brief introduction is provided to demonstrate the importance of fiber-based wearable sensors in personal digital health. Then, the monitoring of biophysical signals through fiber-based sensors is described, and they are classified based on different sensing principles in biophysical signal monitoring (resistive, capacitive, piezoelectric, triboelectric, magnetoelastic, and thermoelectric). After that, the fiber-based biochemical signal sensors are described through the classification of monitoring targets (biofluids and respiratory gases). Finally, a summary is presented on the application prospects and the prevailing challenges of fiber-based sensors, aiming to implement their future role in constructing personal digital health networks.

## 1. Introduction

Fiber materials have a long history of application; their functionalities have evolved from meeting basic survival needs to fulfilling the demand for a higher quality of life in modern society. Recently, fiber-based wearable sensors have emerged as a new application direction [1,2,3,4,5,6] for personal digital health. Personal digital health, including data collection, risk prediction, disease diagnosis, and treatment, utilizes information and communication technology to manage diseases and health risks [7]. The fiber-based wearable sensors have the characteristics of close-range monitoring and portable, non-intrusive monitoring. These characteristics endow them with the ability to collect more accurate and persistent data, which is advantageous in managing diseases and health risks. More importantly, combining fiber-based wearable sensors with machine learning and big data analytics technology can facilitate the construction of a personal digital health network and create an integrated system for personal digital health [8].

**Figure 1:**
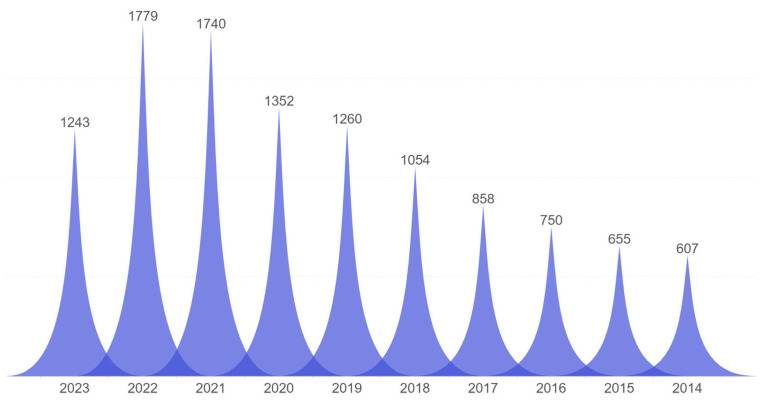
A year-by-year breakdown of the relevant literature. The search criteria employed were the author keywords “fiber sensor”, “yarn sensor”, “textile sensor”, or “fabric sensor”, and the topic “health”. The data used in this analysis were sourced from the Web of Science Core Collection, November 2023.

The primary database for the literature search for this review was the Web of Science Core Collection. The search keywords for the author’s keywords included “fiber sensor” or “yarn sensor” or “fabric sensor” or “textile sensor” and the topic “health”. The document types in this literature search include research papers, review papers, and proceeding papers. The search results indicate that a total of 11,348 relevant documents were retrieved within the ten-year period from 2014 to 2023. Figure 1 clearly illustrates the number of papers published each year related to health and fiber-based sensors. From the trend in quantity, there is an increasing number of relevant papers, especially in the last three years, which have shown a growth rate of approximately 30% compared to the year 2020. This suggests that, with the growing emphasis on personal health, fiber-based sensors for personal digital health systems will have significant application value and market prospects.

Compared to traditional silicon-based sensors and thin film sensors, fiber is a competitive choice for wearable sensors owing to their excellent flexibility, breathability, tailorability, and adaptability to various body parts [9,10,11,12]. The fiber-based sensors can be easily integrated into daily clothing, such as gloves, wristbands, elbow pads, knee pads, and shoe insoles [13,14,15,16]. In addition, the fiber-based sensors can be directly combined with large-area sensing textiles [17,18,19,20]. Products incorporating fiber-based sensors not only allow people to move freely but also ensure that signals from human body deformation can be accurately collected in real time for personal digital health. Such devices are capable of monitoring various types of personal digital health signals, such as biomechanical signals [21,22,23,24,25,26], biotemperature [27,28,29,30], biofluids, as well as respiratory gas [31,32,33,34]. Based on these brilliant advantages and diverse applications, fiber-based sensors show great potential in the fabrication of compact, lightweight, cost-effective, and efficient personal digital health monitoring devices.s

Based on the remarkable performance of fiber-based wearable sensors, a detailed introduction is provided on the recent development of fiber-based wearable sensors for personal digital health monitoring. Firstly, fiber-based sensors for monitoring biophysical signals are introduced and are categorized according to different sensing mechanisms, including biomechanical signal sensing such as resistance-based, capacitance-based, piezoelectric-based, triboelectric-based, and magnetoelastic-based sensing, as well as biotemperature sensing such as resistance-based and thermoelectric-based sensing. Additionally, the classification of fiber-based wearable sensors for monitoring personal biochemical signals is summarized based on different monitoring targets, including biofluids (sweat, saliva, and urine) and respiratory gases (gas composition and gas humidity). Finally, we critically summarize the existing gaps and future challenges for implementing applications of fiber-based wearable sensors in personal digital health.

## 2. Fiber-Based Sensors for Monitoring Biophysical Signals

Biophysical signals are of great significance in the construction of personal digital health systems. For example, monitoring the stability of breathing can prevent respiratory system diseases [35,36]; monitoring foot pressure can achieve gait correction [37,38]; monitoring pulse can assist in diagnosing chronic cardiovascular diseases [39]; and monitoring body temperature can remotely alert for dangerous situations such as a cold or fever [40]. Fiber-based biophysical sensors are capable of detecting a variety of physical quantities in personal digital health, including biomechanical signals generated by human motion and human body temperature signals. Meanwhile, fiber-based sensors can be subtly integrated with daily clothing for adapting to various movements and achieving long-term unobtrusive signal monitoring. These timely signals captured by fiber-based biophysical sensors will be used to construct the personal physical digital health system, liberating people from cumbersome traditional monitoring.

### 2.1. Fiber-Based Biomechanical Signal Sensors

**Figure 2:**
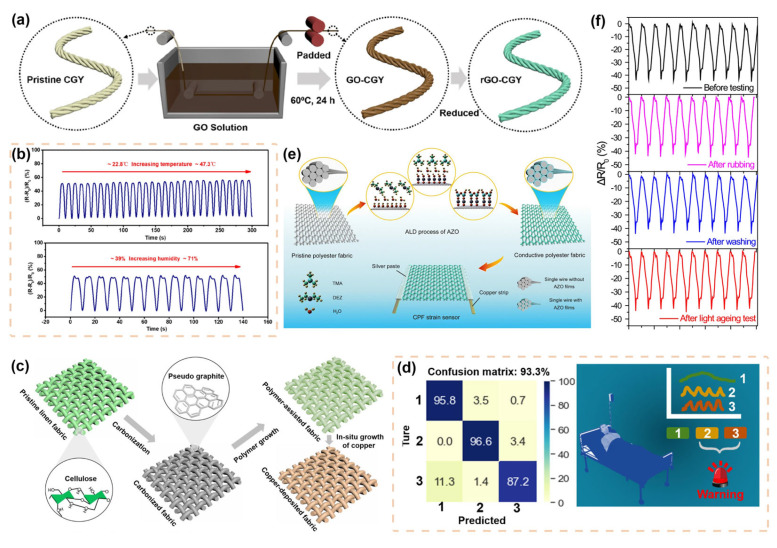
(**a**) Fabrication of conductive treatment of Calotropis gigantea yarn (CGY). (**b**) Demonstrating that the GMF strain sensor is not sensitive to changes in temperature and humidity [45]. Copyright © 2023 Springer Nature. (**c**) The procedure for producing the multi-pathway conductive fabrics. (**d**) The confusion matrix for distinguishing different breathing patterns and the schematic diagram of the respiratory monitoring system [46]. Copyright © 2021 Elsevier. (**e**) Illustration of the CPF strain sensor prepared based on ALD. (**f**) Demonstration of the CPF strain sensors maintaining excellent durability under the effects of friction, washing, and light aging [47] Copyright © 2019 Springer Nature.

Many attractive breakthroughs in fiber-based biomechanical signal sensors were made for personal digital health, including resistive sensors, capacitive sensors, piezoelectric sensors, frictional sensors, and magnetoelastic sensors. Fiber-based resistive sensors have gained widespread attention because of their convenient fabrication process, simple construction, and high sensitivity. The mechanism of fiber-based resistive sensors refers to the conversion of mechanical signals applied to the human skin to changes in electrical resistance, and they can be divided into resistive strain sensors [41,42] and resistive pressure sensors [19,20]. Currently, fiber-based resistive strain sensors have been intensively studied [25,43,44]. For example, by coating graphene oxidized (GO) onto the calotropis gigantea yarn via the pad-dyeing method (Figure 2a) and then interweaving it with polyurethane yarn through a weaving process, a highly breathable and sensitivity-tunable graphene-modified fabric (GMF) strain sensor was fabricated [45]. The GMF strain sensor exhibits stable detection performance in varying temperature and humidity environments in Figure 2b. This is indispensable for fiber-based sensors to monitor personal digital health data in complex human movements and dynamically changing microclimate systems. Figure 2c illustrates the carbonization process to treat flax fabric and then further enhances its conductivity by depositing highly conductive copper particles with the assistance of the (NH_4_)_2_PdCl_4_ polymer [46]. This resulted in the production of an outstanding performance strain sensor with high sensitivity (gauge factor (GF) ≈ 3557.6 in the strain range of 0–48% and GF ≈ 47.8 in the strain range of 48% to 150%. For strain sensors, GF refers to the ratio between the rate of resistance change and strain.) and they have a wide strain monitoring range (up to 300% strain). Moreover, the authors combined it with a convolutional neural network model to develop a healthcare system for personal respiratory monitoring, aiming to accurately detect and differentiate various respiratory patterns. After training, the system achieved an average classification accuracy of 93.3% for three monitored targets: normal respiration, rapid respiration, and cough (Figure 2d). This is of great significance for real-time monitoring and intelligent warning of patients’ respiratory patterns, which can assist in building a personal digital health monitoring network.

**Figure 3:**
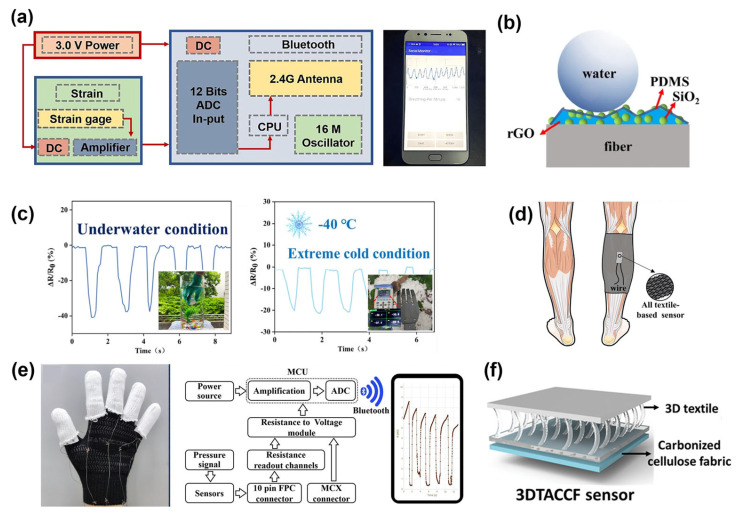
(**a**) Schematic diagram of the developed wireless signal processing module and real-time display interface for smartphones [48]. Copyright © 2023 Elsevier. (**b**) Detection of the signals generated by the strain sensor under extreme conditions. (**b**) Schematic of SiO_2_ and PDMS-modified treated fibers. (**c**) Demonstrate the ability of strain sensors to monitor underwater and extreme cold conditions. The insets are the photographs of the sensor in operation [49]. Copyright © 2022 Springer Nature. (**d**) Schematic diagram of the fiber-based pressure transducer used to monitor the amount of pressure applied to a varicose vein treatment band. (**e**) A photo of the smart glove and a schematic of the transfer to the smart device app via Bluetooth technology [13]. Copyright © 2023 Elsevier. (**f**) Schematic structure of a wearable pressure sensor prepared through 3D textile-assisted carbonized cellulose fabric (3DTACCF) [52]. Copyright © 2022 Elsevier.

In addition to the positive resistive effect, negative resistive fiber-based sensors have also been intensively studied. As shown in Figure 2e, atomic layer deposition (ALD) technology was used to fabricate a negatively resistive conductive polyester fabric (CPF) strain sensor based on a woven structure [47]. With the advantage of the ALD forming stable chemical bonds, Figure 2f demonstrates the excellent durability of the CPF strain sensor under long-term washing for 200 min, reciprocating friction of 50 kPa, and accelerated aging by light exposure. The excellent anti-interference performance is crucial for maintaining data consistency in long-term digital health monitoring using fiber-based sensors. Liu et al. used 3-glycidyloxypropyl trimethoxy silane-assisted graphene to modify silk/polyurethane composite yarns, leading to the fabrication of wearable strain sensors [48]. This sensor exhibits a highly linear negative resistive effect (R^2^ = 99.8). In addition, Figure 3a shows the potential application of a body area sensor network constructed by the sensor and wireless signal transmission system in personal digital health management. The human body is at a higher risk of being compromised when exposed to extreme environments. Therefore, the development of fiber-based sensors that can be applied underwater, in high temperatures, extreme cold, and other conditions to monitor personal digital health signals is an important direction. In Figure 3c, the sensor exhibits superhydrophobicity ability due to the modified treatment of SiO_2_ and PDMS (Figure 3b), which improves their durability and extends their applicability in extreme environments, such as underwater conditions and extreme cold conditions [49]. However, the addition of additional PDMS coatings has an effect on the moisture absorption and air permeability of fiber-based sensors, and a balance has to be found between the high additional performance and comfort of the sensors in subsequent studies.

Developing pressure sensors based on resistive mechanisms is one of the most popular approaches. The resistive pressure sensor can convert the geometric changes caused by external pressure signals into resistance variations [50]. In one study, a pressure sensor (with a low detection limit of 1 Pa and a wide detection range of 0–470 kPa) based on regenerated fiber fabrics was fabricated using the carbonization method, demonstrating its potential for application in next-generation wearable electronic devices [51]. The sensor proved to be effective in detecting weak airflow, wrist pulses, and sound signals. Liu et al. developed a flexible pressure sensor using a two-layer composite fabric that combines highly elastic Venetian fabric and non-elastic polyester [13]. Carbon nanotubes (CNTs) were embedded in the upper layer of Venetian fabric using an electrostatic layer-by-layer assembly technique and used as a sensing layer. Subsequently, flexible interconnecting electrodes were printed on the lower polyester fabric using a screen printing technique, and the upper and lower layers were conveniently sewn together to prepare an all-fabric pressure sensor. With the advantage of the textile’s outstanding wearability and flexibility, the fiber-based sensor can be conveniently integrated into elastic socks used by patients with venous insufficiency to monitor the applied pressure. The results demonstrate the sensor can clearly distinguish the applied pressure (Figure 3d), highlighting the potential of fiber-based sensors in digital health monitoring for medical assistance. In addition, the fiber-based pressure sensors can be flexibly assembled in gloves with Bluetooth transmitters (Figure 3e), and real-time pressure signals could be observed in a smartphone app, helping to build a personal pressure-sensing digital health network system. By taking advantage of the 3D knitted fabric structure, a wearable pressure sensor with high sensitivity (32.13 kPa^−1^) was manufactured (Figure 3f) [52]. The prestrained monofilaments within the fabric structure serve as the pressure transmission structure, enhancing the local strain within the sensor. The sensitivity of the sensor can be adjusted by modulating the length and density of individual fibers, demonstrating the advantages of the ingenious structure of fiber-based sensors in constructing pressure sensors and serving as a new-generation wearable sensor substrate for personal digital health sensors.

**Figure 4:**
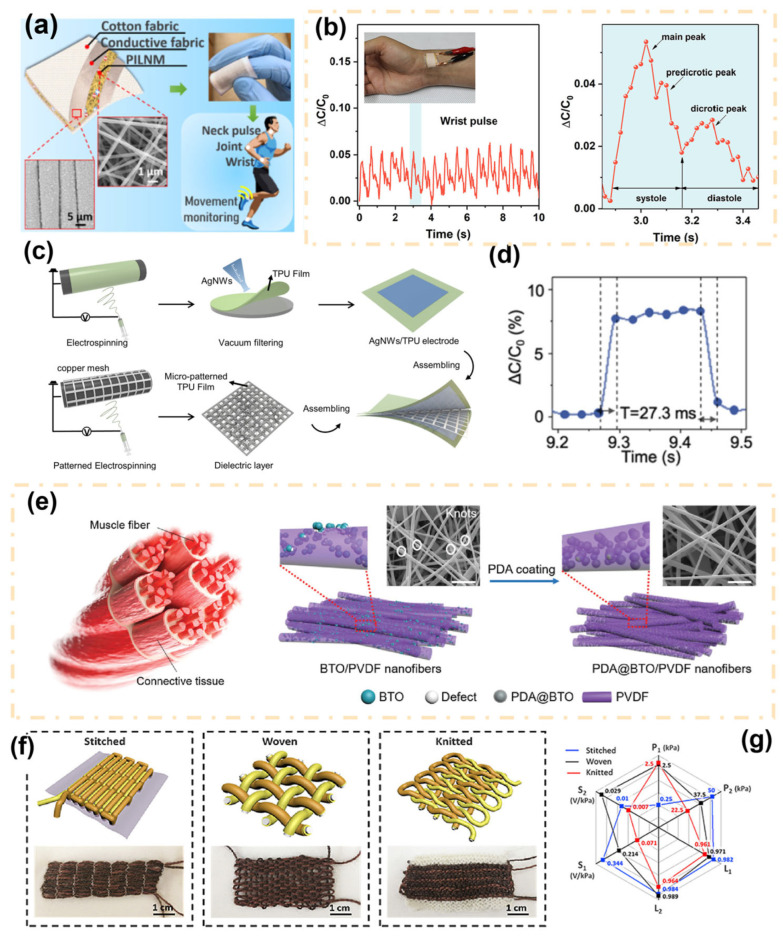
(**a**) A water-washable capacitive pressure sensor was prepared based on hydrophobic nanofiber membranes [54]. Copyright © 2019 American Chemical Society. (**b**) The electrical output curve of the sensor applied to human pulse monitoring [55]. Copyright © 2021 American Chemical Society (**c**) The schematic diagram of capacitive pressure sensors based on micropatterned dielectric layers. (**d**) Demonstration of the fast response/recovery time of micropatterned capacitive pressure sensors [56]. Copyright © 2021 American Chemical Society. (**e**) The illustration of a piezoelectric nanofiber sensor inspired by muscle fibers [60]. Copyright © 2021 Wiley–VCH. (**f**) Photos of the textile triboelectric nanogenerators with sewn, woven, and knitted structures. (**g**) Effect of different fabric structures on transition point pressure, sensitivity, and linearity of the sensor [61]. Copyright © 2020 Elsevier.

The capacitive fiber-based sensor represents another commonly employed method for fabricating pressure sensors. It mainly consists of the top electrode, bottom electrode, insulator, and substrate. Fiber-based capacitive sensors can react to the mechanical force applied to the human body through the change in capacitance, which is caused by the variation in the gap between plates or the frontal area. Fiber-based capacitive pressure sensors are known for their flexibility, comfort, breathability, and durability, making them an excellent sensor type for personal digital health monitoring [53]. However, it still faces a dilemma with maintaining stability with the effects of environmental changes. Wang et al. presented a water-washable capacitive pressure sensor based on a hydrophobic poly(ionic liquid) nanofibrous membrane (PILNM) (Figure 4a) [54]. Poly(1-butyl-3-vinylimidazolium bis(trifluoromethane sulfonyl)imide) ([PBVIm] [TFSI]) was added as the main ion-conductive component in the electrospinning solution. This component contains a large number of polarizable ions, enabling the formation of a pressure sensor with high capacitance and sensitivity. Moreover, because of the insolubility of the [PBVIm][TFSI] in water, the fabricated sensor showed high stability under high humidity conditions (70% relative humidity) and repeated washing (more than 10 machine washing cycles).

Linearity, sensitivity, and response time of fiber-based capacitive sensors are among the basic performance indicators. A method using hybrid ionic nanofiber membranes is proposed to improve the sensitivity and linear range of capacitive pressure sensors [55]. This membrane consists of MXene, an ionic salt of lithium sulfonamides, and a poly(vinyl alcohol) elastomer matrix. In the absence of pressure, the MXene surface on the functional layer forms hydrogen bonding pairs with ions that remain confined to the MXene surface, inhibiting the formation of the capacitive layer. When external pressure is applied, these ion pairings dissociate from the MXene surface, and a thick capacitive layer is formed at the contact interface through an ion pumping process, leading to a significant change in capacitance and enabling highly sensitive pressure sensing. The sensor exhibits high sensitivity (5.5 kPa^−1^ and 1.5 kPa^−1^ in the linear range of 0–30 kPa and 30–250 kPa) and fast response time (70.4 ms). These excellent performances give it the potential to serve as a new electronic skin for constructing personal digital health networks. Figure 4b shows that the prepared sensor can not only distinguish the pulse rate but also display prominent characteristic peaks, which can be applied to the monitoring of diseases such as arrhythmia. Figure 4c illustrates a highly sensitive, ultra-thin, all-fabric capacitive pressure sensor based on a breathable micropatterned dielectric layer [56]. The regular gaps formed by the micropatterns effectively increase the compressibility and dielectric constant. It can be seen from Figure 4d that the response and recovery time of the sensor are significantly reduced because the high dielectric constant of the sensor could reduce the viscoelasticity of the device. Fast response and recovery times mean that the sensor can capture and respond to various health data from the human body in real time, which is crucial for effective personal digital health monitoring.

Resistive and capacitive fiber-based sensors rely on external power sources to operate, limiting their portability in practical personal digital health monitoring applications. Piezoelectric sensors, on the other hand, are self-powered sensors that can actively convert mechanical pressure into voltage/current signals without the need for an additional power source, showing great potential in human health monitoring [57,58]. The sensing mechanism of piezoelectric fiber-based sensors involves compression and deformation of the sensor due to external pressure, causing polarization within the material, which generates a positive current between the two electrodes through the external load. When the external pressure is removed, the system recovers due to the elasticity of its initial shape, producing an alternating short-circuit current [59].

Poly(vinylidene fluoride) (PVDF) and its copolymers are widely used because of their excellent piezoelectric and excellent chemical, mechanical, and thermal properties [62]. Li et al. prepared PVDF-based nanofiber membranes by electrospinning. They investigated the influence of multi-walled carbon nanotubes and BaTiO_3_ as additives in the spinning solution on the sensor performance [63]. The CNTs and BaTiO_3_ acting as nucleating agents promote the formation of more β phases in PVDF, thus increasing the dielectric constant. The fabricated piezoelectric sensors showed excellent sensitivity (up to 116 mV·kPa^−1^ in the range of 0–10 N) and durability (over 12,000 cycles). Due to different contact areas and weights, common laboratory tools applied to piezoelectric sensors can clearly display different voltage output signals, which illustrates their potential application prospects in electronic skin. Combining piezoelectric ceramics and piezoelectric polymers to form piezoelectric composites can enhance the performance of piezoelectric sensors. Based on this, polydopamine (PDA) particles were dispersed into BaTiO_3_/PVDF nanofibers (Figure 4e) [60], effectively promoting the physical contact and stress transfer capability between the inorganic filler and organic matrix in the composite fibers. As a result, the mechanical strength and electromechanical coupling efficiency of the piezoelectric composite fibers were significantly improved (the introduction of 3.02 wt% PDA doping increased the piezoelectric performance by 47%). The prepared high-performance piezoelectric nanofiber sensor proves its great potential in personal digital health systems, including active human motion monitoring and pulse recognition.

A triboelectric nanogenerator (TENG) is another type of self-powered sensor. Its working modes can be broadly divided into four types: vertical contact-separation mode, lateral sliding mode, single electrode mode, and independent friction mode. Taking the TENG in the contact-separation mode as an example, it generates frictional charges on the surface when two different materials come into contact and establishes a potential difference upon separation. This leads to the generation of transient current and eventually achieves equilibrium when completely separated on the surface [64]. The TENG has plenty of advantages, such as a simple structure, high energy conversion efficiency, and a high output voltage. It provides a novel energy harvesting and monitoring method for wearable health devices [65,66,67].

**Figure 5:**
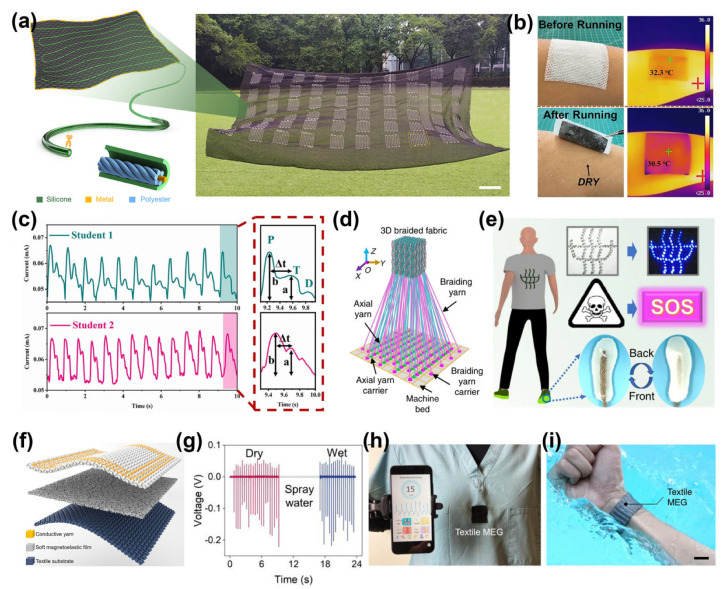
(**a**) The photo shows an active sensing bedsheet with a large area prepared through functional fibers and its actual application. Scale bar: 10 cm [17]. Copyright © 2020 Elsevier. (**b**) Demonstrating the ability of DMWES to keep skin dry and cool. (**c**) Demonstrating that the DMWES can discriminate pulse signals [68]. Copyright © 2023 Springer Nature. (**d**) Schematic of the 3D knitting process. (**e**) A smart insole prepared by a triboelectric nanogenerator for active sensing, illuminating warning lights, and sending danger signals [37]. Copyright © 2020 Springer Nature. (**f**) Schematic structure of the textile MEG. (**g**) Demonstration of the intrinsic waterproofness of the textile MEG. (**h**) Photograph of the textile MEG used for respiratory monitoring in the field as well as respiratory sensing curves [69]. Copyright © 2021 Elsevier. (**i**) The textile MEG can be used directly for underwater motion monitoring. Scale bar: 6 cm [14]. Copyright © 2021 Springer Nature.

The fiber-based TENGs prepared by textile technology can be conveniently applied to everyday clothing. Figure 4f displays the fabricated TENGs with different textile structures (stitched, woven, and knitted) [61]. In this work, the performances of fiber-based sensors between three different structures were explored, including sensitivity, operating range, linearity, and water-washing durability. It shows that the sewn TENG has a higher sensitivity due to the larger contact area between the yarns, with an initial sensitivity of 0.311 V/kPa. The TENG with woven structure possesses the best washability after washing 20 times, which benefits from the tight interweaving of yarns. It appears the TENG, with its woven structure, is the best candidate for use as a washable textile pressure sensor. The authors comprehensively combined the transition point pressure, sensitivity, and linearity of different regions in Figure 4g, where P represents the transition point pressure and S and L represent the sensitivity and linearity of the first and second regions, respectively. The performance differences brought by these different textile structures are crucial for researchers to choose the appropriate textile structure to design a new generation of personal digital health monitoring networks. The monitoring of personal digital health signals during the night is also crucial. The smart bedsheet designed by Zhou et al. can effectively monitor sleep positions, the number of turns, and respiratory rate during the night [17]. The washable smart fiber sensors in the bedsheet are prepared by wrapping conductive yarn cores with ultra-thin silicon fibers. Through the combined effect of frictional charging and electrostatic induction, it actively converts pressure changes into electrical signals. The authors integrated the sensor into regular bed sheets (Figure 5a) to achieve smart monitoring of sleeping posture. The fiber-based sensor can also be combined with subtle respiration and electrocardiogram signals to collect real-time data, provide timely warnings, and facilitate the detection of potential sudden illnesses during sleep. By designing different hydrophobic-hydrophilic gradients, an electronic skin with directional moisture-wicking electronic skin (DMWES) was designed [68]. The surface energy gradient and capillary effect of the sensor enable the automatic absorption and transfer of sweat from the skin. Specifically, the sensor consists of a hydrophobic layer formed by the CNT-modified PVDF nanofibers and a hydrophilic layer constituted of polyacrylonitrile (PAN) nanofibers. As shown in Figure 5b, the hydrophobic layer prevents sweat from entering, while the hydrophilic layer absorbs sweat and facilitates rapid evaporation, ensuring dry and cool skin during sensor usage. This is vital for maintaining comfort during long-term personal digital health monitoring. The integration of resistive pressure sensors and TENG sensors into the DMWES enables comprehensive health perception and biomechanical energy harvesting. For example, it can be integrated into shoe insoles to monitor gait signals or incorporated into clothing to monitor pulse signals. In Figure 5c, the DMWES can identify changes in the frequency and intensity of pulse before and after exercise and can distinguish typical peaks in the pulse waveform (P-wave, T-wave, and D-wave).

The TENGs with 3D structures have larger contact-separation forces during usage, thereby enhancing their output capabilities. For instance, a 3D knitted fabric using a double-needle bed knitting machine was designed as a self-powered bending/pressure sensor [70]. Compared with woven fabrics, knitted fabrics with plain weft and warp structures exhibit better flexibility and breathability while effortlessly collecting energy from human motion. Similarly, a 3D-woven TENG using a homemade 3D weaving machine with the four-step weaving technique is exhibited in Figure 5d [37]. A wireless smart insole system was created to monitor human motion and record electrical digital health signals. A self-developed data processing program based on LabVIEW allows for real-time calculation and display of signals such as step count, average speed, and exercise intensity. In addition, the electrical energy generated during motion can illuminate light-emitting diode (LED) safety indicators and remotely send distress signals in potentially dangerous situations (Figure 5e).

Piezoelectric and triboelectric effects can convert mechanical energy from the body into electrical energy for human digital health monitoring. However, the generated electrical current relies on the transfer of electric dipoles at the material interface, which is susceptible to the influence of sweat and environmental humidity. A straightforward and effective method is to use elastomer to encapsulate fiber-based sensors, but it can lead to a reduction in the comfort and breathability of textiles. Hence, it is imperative to pursue moisture-resistant fiber-based sensors. Recently, a design method for a fiber-based sensor that is completely unaffected by moisture was proposed [69]. This approach is primarily based on the giant magnetoelastic effect in soft materials, and it paves the way for further design of a fiber-based magnetoelastic generator (MEG) for the conversion of biomechanical energy. It is assembled from a three-layer textile structure consisting of a soft magnetic elastic membrane, textile coils, and textile substrates, as illustrated in Figure 5f. The MEG enables a three-step conversion of mechanical-magnetic flux into electrical energy. Due to its immunity to magnetic field influence in high humidity and even liquid environments (Figure 5g), the MEG does not require any encapsulation and possesses waterproof capabilities. It can be conveniently used as a self-powered sensor and is suitable for monitoring the body’s chest, where sweat production is high. In addition, the MEG, combined with Bluetooth data transmission and machine learning technologies, can monitor respiration and distinguish breathing patterns in real-time (Figure 5h). This has significant implications for personal digital health development. Another work reported the magnetoelastic effect in one-dimensional soft fibers and utilized the coupling of the magnetoelastic effect with magnetic induction by weaving one-dimensional soft fibers with conductive yarn to fabricate a MEG [14]. The MEG was applied to wearable fabrics to convert pulse signals into electrical signals. In Figure 5i, the wristband integrated with the prepared MEG can complete real-time detection of pulse beating during underwater activities without any packaging. The magnetoelastic effect in soft materials offers a new avenue for the stable monitoring of personal digital health signals in dynamically changing environments.

### 2.2. Biological Temperature Sensor

Human body temperature is a dynamic process, and the change in body temperature can reflect a series of physiological conditions such as fever, coldness, blood flow rate, muscle fatigue, etc. Fiber-based wearable temperature sensors with high sensitivity, great accuracy, and fast response can effectively guard against physical abnormalities and keep people away from the discomfort caused by body temperature changes. Meanwhile, they can meet the requirement of wearing owing to excellent comfort and breathability. Therefore, it is of significance to develop fiber-based wearable temperature sensors to monitor body temperature and establish an analysis system for building personal digital health systems [71,72,73,74].

**Figure 6:**
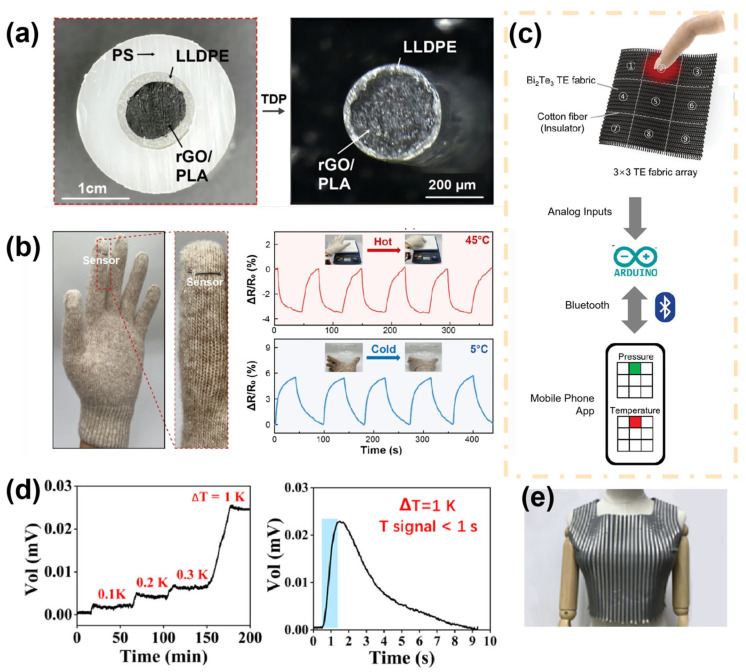
(**a**) Optical photo of fiber cross-section before and after the thermal drawing process, with PLA and rGO forming a sensing layer. (**b**) Photograph and sensing curves of a temperature sensing glove [27]. Copyright © 2023 Springer Nature. (**c**) A sensor array capable of simultaneously detecting temperature and pressure stimuli can be transmitted to smart devices via Bluetooth [78]. Copyright © 2023 Wiley-VCH. (**d**) The voltage output curve of the PPSF sensor, demonstrates its high resolution and fast response. (**e**) Photograph of a vest made directly from the PPSF material [11]. Copyright © 2020 American Chemical Society.

The application of fiber-based sensors in biological temperature monitoring focuses primarily on two aspects: resistive sensing and thermoelectric sensing. The thermal resistance effect refers to the change in resistance with temperature. Figure 6a illustrates a three-layer nanocomposite fiber temperature sensor prepared via the thermal drawing process, where the innermost layer used as a temperature sensing layer is composed of polylactic acid (PLA) doped with reduced graphene oxide (rGO) [27]. The electrical conductivity of rGO is positively correlated with temperature, leading to a decrease in resistance of the fiber temperature sensor as the temperature increases. The motion glove integrated with a fiber-based temperature sensor has a sensitivity of −0.285%/°C, and it demonstrates sensing effects in external environments of 45 °C and 5 °C (Figure 6b). However, its lower sensitivity resulted in inadequate monitoring performance within the human body’s temperature range. Chen et al. fabricated nanofiber membranes based on ionic liquid ([EMIm][NTf_2_]) and thermoplastic urethane using electrospinning technology. These nanofiber membranes exhibit high sensitivity to both strain (GF ≈ 1 within the 0–10% strain range) and temperature (2.75%/°C within the 30–40 °C temperature range), as well as great linearity (0.998 within the temperature range of the human body), making them suitable for dual-modal sensing [75]. However, the multifunctional detection performance of the sensor may be distorted by mixed stimuli, leading to distorted detection results.

Thermoelectric sensors generate electrical current or voltage in response to temperature changes. This phenomenon can be used to create generators that use variations in human body heat to generate electricity and transmit temperature signal changes based on variations in current or voltage [76,77]. For instance, a versatile stretchable fabric sensor based on Bi_2_Te_3_ was fabricated by using a simple chemical reduction method that could generate an electrical current in response to temperature changes. In addition, they designed a 3 × 3 sensor array system capable of simultaneously detecting pressure and temperature changes (Figure 6c) [78]. Li et al. used 3D spacer fabrics as the substrate and then modified them with poly(3,4-ethylenedioxythiophene-poly styrene sulfonate) polymer to prepare a large-area wearable self-powered pressure-temperature sensor (PPSF). As shown in Figure 6d, the temperature response resolution of the sensor can reach 0.1 K with a response time of less than 1 s. This dual-functional sensor can output independent voltage/current signals under dual stimulation of temperature and pressure, thus avoiding interference from mixed stimulation. The intermediate layer of the 3D spacer fabric contains a large amount of insulating stagnant air, which can reduce temperature interference on both sides of the sensor, thereby outputting stable voltage signals. Moreover, Figure 6e shows that the PPSF can be easily tailored and further used to create a large-area sensing electronic textile vest without the need for embedding in the fabric by sewing or other methods [11]. This offers advantages for the preparation of integrated personal digital health monitoring networks.

In conclusion, the application of fiber-based biomechanical sensors to monitor the real-time movement status and temperature of the human body has made great progress. Sensors with various monitoring principles can achieve accurate, sensitive, and long-term monitoring of digital health signals in an individual. In addition, they have validated the effectiveness of fiber-based sensors in combination with digital technologies through various methods. However, as the functional requirements for the monitoring of mechanical signals used for personal digital health increase, it is not sufficient to realize only unimodal (single strain, bending, compression, friction, etc.) monitoring, and therefore large-area multimodal monitoring of the whole body area becomes necessary. In addition, it is potentially difficult to integrate the sensor system with a real garment. Typically, fiber-based sensors are lightweight and flexible, but the wires and signal-processing devices attached to them are difficult to integrate into garments, requiring more effort to develop flexible circuit technology.

## 3. Fiber-Based Sensors for Monitoring Biochemical Signals

As a significant indicator of the body’s internal state, biochemical signals carry a plethora of health information for individuals. For example, the concentration of sodium ions can indicate an electrolyte imbalance in the body. A higher concentration indicates a lower blood concentration, while excessively high concentrations may indicate dehydration or hyponatremia [79]. By monitoring these biochemical signals, we can understand key indicators of an individual’s health status, metabolic level, disease risks, etc. Fiber-based biochemical sensors are designed to monitor individuals’ biochemical signals, including biological fluids (such as sweat, saliva, and urine) as well as respiratory gases. The monitored data can be collected and analyzed by integrating the sensors with mobile smart devices, providing immediate health management, medical diagnosis, and treatment for personal digital health.

### 3.1. Fiber-Based Biofluid Signal Sensors

The key components of biofluidic signaling are sweat, saliva, and urine, where sweat contains a variety of biomarkers such as glucose, lactate, cortisol, and ions that can be used to indicate the health status of the body [80,81,82,83]. Fiber-based sweat sensors can adhere closely to the skin and adapt to skin movement, providing comfort, breathability, and accuracy, which is highly beneficial for monitoring human sweat.

**Figure 7:**
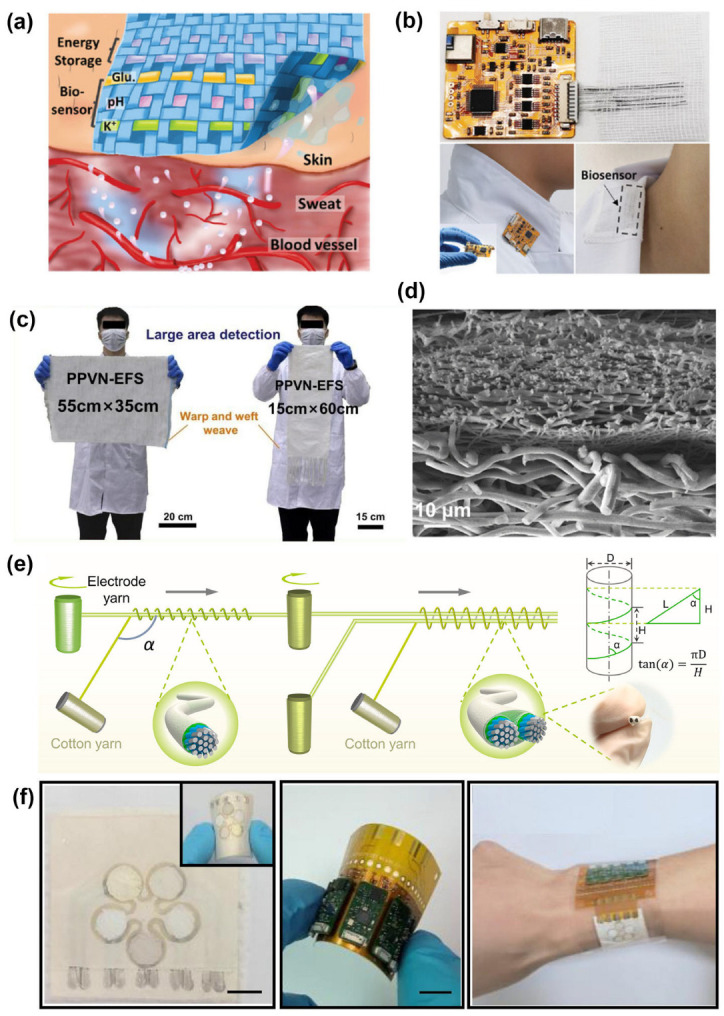
(**a**) The illustration of multiple sensing yarns integrated into a fabric patch, and (**b**) photographs of flexible printed circuit boards and sensors attached to clothing [87]. Copyright © 2023 Wiley–VCH. (**c**) Photograph of the large-scale electrochemical fiber-based sweat sensor [18]. Copyright © 2023 Elsevier. (**d**) Increasing the sensing efficiency of the modified Janus fabrics to about 10 times that of the unmodified ones [88]. Copyright © 2023 Royal Society of Chemistry. (**e**) The fabrication process of the bi-electrode structure of the SYBSC [31]. Copyright © 2023 Elsevier. (**f**) The photos of the NFMAS in the flat and bent state, the wireless flexible circuit board, and worn on the wrist [89]. Copyright © 2023 Elsevier.

The use of electrochemical sensors to monitor biomarkers in human sweat is a common method [84,85,86]. For instance, a multifunctional textile sensor made of rGO/polyaniline (PANI) hybrid fibers can be used to monitor multiple biomarkers simultaneously [87]. Taking advantage of the excellent charge storage capacity of polyaniline in bioelectrolyte solution, the authors assembled two fiber electrodes into a fiber-based supercapacitor with an obvious linear relationship with pH change. In addition, K^+^ selective membranes and glucose oxidase were used to monitor K^+^ and glucose. Then, the multifunctional sweat sensor was integrated into a fabric patch by the weaving method (Figure 7a). To illustrate the efficacy of this fiber-based sweat sensor, a flexible integrated circuit module for analysis and data transmission was developed (Figure 7b) to monitor human sweat digital signals, enabling real-time display of biomarker concentrations in sweat on smart devices.

The absorption of sweat by ordinary fabrics will reduce the diffusion of sweat to the sensing area, hindering rapid and accurate monitoring. To overcome this challenge, Mo et al. incorporated warp and weft yarns with remarkable hydrophilic differences to manufacture large-scale electrochemical textile sweat sensors (Figure 7c) [18]. The warp yarn was made of superhydrophobic polyester fibers, whereas the weft yarn, which contained valinomycin, functioned as the sensing thread. Valinomycin is used as a selective carrier for K^+^ ions to achieve rapid response (2.1 s) and long-term stability (maintaining stable electrical signal output for over 6000 s in a 4 mM KCl solution) for the K^+^ ions in sweat. Alternatively, a bilayered nanofiber membrane was used to facilitate vertical transport of sweat (Figure 7d) [88], which could reduce the ineffective diffusion of sweat and shorten reaction time. For the sensing fabric with asymmetric wetting properties and porosity gradients, the skin-facing layer of the bilayer electrospun fiber membrane exhibits high hydrophobicity, while the upper modified region acquires hydrophilic groups through selective oxygen plasma treatment. Sweat was transported against gravity from the skin surface to the modified region and concentrated on the modified surface, which contains a highly efficient catalyst (Cu_2_O). It endows the fabric with the functions of directional sweat collection, reduces the ineffective diffusion of sweat, shortens the response time, and improves sweat collection efficiency tenfold. The ingenious structure of fiber-based sensors enables efficient sweat collection and sensing capabilities, which is of great significance for building a digital health network.

The high ion content of sweat allows it to be used as a natural electrolyte for preparing biocompatible energy sources. Xiao et al. developed a sweat-driven yarn-based bio-supercapacitor with a symmetrical dual-electrode structure (Figure 7e) [31]. Stainless steel fiber-based yarn, conductive polymer, and sweat were used as the current collector, active material, and electrolyte, respectively. Cotton fibers were coated on the outer layer to achieve the dual-electrode structure and enhance moisture absorption. This sweat-activated self-charging system can power wireless analyzers to construct integrated sweat-sensing textiles. It collects sweat during physical activity to charge the capacitors and releases current to power miniaturized sweat pH monitors after switching on, which then transmit real-time data to smartphones for display. Colorimetric sensing is also a commonly used method for measuring biomarkers in sweat. In Figure 7f, a petal-shaped nanofiber-based microfluidic analysis system (NFMAS) was developed [89]. The NFMAS can spontaneously capture sweat and transport it to different sensing chambers through microchannels, which can be used to simultaneously detect the levels of glucose, lactate, pH, Cl^−^, and urea in sweat. The changes in a color’s RGB values are transmitted to a smartphone through the integrated NFC chip, eliminating the need for visual estimation using color cards and providing more accurate analysis results. Although wearable fiber-based sweat sensors show many advantages for detecting biosignals, such as non-invasiveness, real-time monitoring, and wearability, there are still some challenges at the current stage. The sensor can only collect sweat during physical activity, which restricts the time it can be used. Therefore, some designs have only been tested using synthetic sweat [90,91]. Furthermore, the concentration of biomarkers in sweat is lower compared to blood, demanding higher sensitivity and accuracy in detection.

Saliva is a readily available biofluid that contains important biomarkers, which can indicate the health status of the human body [92,93]. Glucose in saliva is a good example, as it shows a strong correlation with its concentration in blood. Compared to traditional blood testing methods, which can be invasive, saliva-based testing is a highly promising alternative [94]. Xu et al. fabricated a CuO nanoparticle-modified poly(caprolactone) (PCL)@polypyrrole (PPy) fiber-modified indium tin oxide electrode (referred to as CuO/PCL@PPy/ITO electrode) by a combination of electrospinning and electrodeposition methods for non-enzyme detection of glucose in saliva. The electrode, owing to the synergistic effect between CuO and the PPy and the unique three-dimensional network structure of electrospinning, offers good selectivity, repeatability, and a high catalytic surface area for the sensor. The CuO/PCL@PPy/ITO electrode exhibits a linear electrochemical response curve for glucose concentration in the range of 0.002–6 mM, which accurately reflects the glucose content in human saliva. To enhance the linear sensitivity of CuO to glucose, a nanohybrid array platform for non-enzyme glucose sensing was established using a copper wire substrate with a core-shell structure [95]. The core-shell structure facilitates the diffusion of ions and molecules from the shell to the core region and exposes numerous electroactive sites for catalytic processes, thereby enhancing the sensing sensitivity. Compared to the saliva glucose sensing electrode prepared by Xu et al. with a sensitivity of approximately 1500 A mM^−1^cm^−2^, this one-dimensional core-shell fiber electrode exhibits a high sensitivity of 2445 A mM^−1^cm^−2^ and a fast response time of 1.62 s [96]. Although the accurate and rapid detection of biomarkers in saliva through fiber-based sensors offers a promising non-invasive approach to human health assessment, there is a certain development space in constructing personal digital health systems for practical applications. Factors such as the method of saliva collection (active or passive), age differences among subjects, and dietary habits may affect the actual content of biomarkers in saliva. Moreover, the correlation between biomarker levels in saliva and blood still requires further investigation.

**Figure 8:**
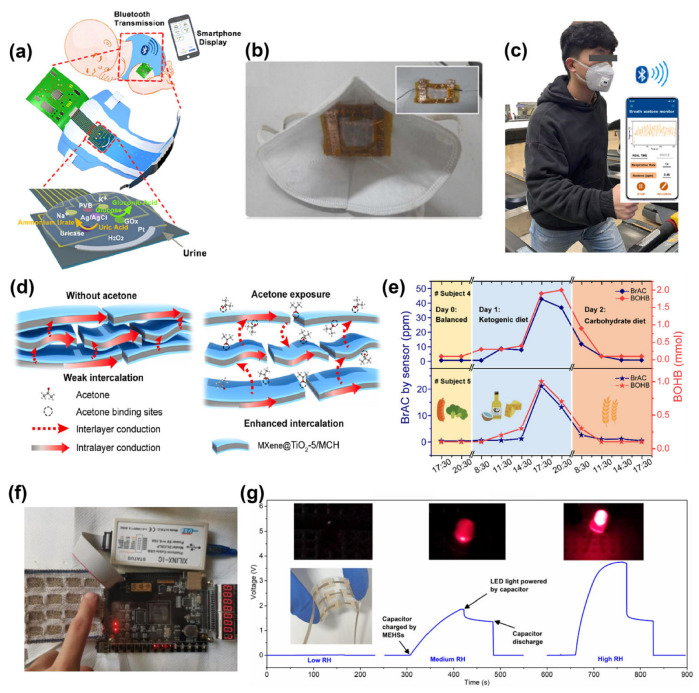
(**a**) Schematic of the MECS integrated with cotton diapers [100]. Copyright © 2022 American Chemical Society. (**b**) A photo of an N95 mask integrated with TENG, which is used to power the internal ammonia sensor [107]. Copyright © 2023 Elsevier. (**c**) Photograph of wearing the acetone sensor while exercising. (**d**) Working mechanism of the acetone sensor. (**e**) Acetone sensor masks for measuring the dynamic respiratory acetone level of volunteers under different dietary patterns. Blood β-hydroxybutyrate (BOHB) is used to assist in validating the data accuracy of acetone sensors [108]. Copyright © 2023 Elsevier. (**f**) A wearable fabric keyboard with humidity as the stimulus signal [109]. Copyright © 2021 Elsevier. (**g**) Time-voltage curves of the MEHS sensor for driving the LED illumination, with an illustration of the circuit schematic diagram [110]. Copyright © 2022 Elsevier.

Urine has the characteristics of a large excretion volume and easy collection. The concentration of biomarkers in urine can reflect various diseases of the urinary system, such as kidney diseases, diabetes, and electrolyte disorders [97,98]. Using sensors to detect the concentration of biomarkers is an effective method to help humans stay healthy. The previous work reported a flexible, self-powered biosensor used to detect glucose in urine that can be easily integrated into a diaper [99]. This sensor utilizes glucose in urine as fuel to drive an enzymatic biofuel cell (EBFC), which then outputs electricity to a power management device with capacitors to drive the LED. The concentration of glucose is directly proportional to the flashing frequency of the LED in the range of 1–5 mM (r = 0.994). In the EBFC, glucose oxidase and MnO_2_ are used as a bioanode and a bio-cathode. They also used a honeycomb hexagonal electrode array to enhance the power generation efficiency, achieving a maximum power density of 220 W cm^−2^ at 5 mM glucose. Li et al. developed a smart diaper integrated with multiplex electrochemical sensors (MECS), which can simultaneously monitor multiple parameters such as K^+^, Na^+^, reactive oxygen species, uric acid, and glucose in urine. As shown in Figure 8a, the captured signals can be wirelessly transmitted to smart devices via a flexible printed circuit board with a Bluetooth module [100]. However, the application of the sensors in the diaper is only in the laboratory stage due to the unstable functionality of combining the flexible circuit board with the sensors and the requirement of an external power source. Continuous improvements in flexible circuit technology will be required in the future to be truly applied to commercial diapers.

### 3.2. Fiber-Based Respiratory Gas Sensor

To date, more than 3000 volatile organic compounds (VOCs) have been identified in human exhaled breath. Among them, dozens of VOCs can serve as respiratory biomarkers, providing important information about metabolic disorders or functional impairments in the human body [101,102,103,104]. Patients with pathological conditions have specific volatile organic compounds in their respiratory gases, and monitoring these compounds can assist in maintaining human health. For example, the concentration of ammonia in human breath is associated with kidney disease, and the concentration of ammonia in the breath of patients is about five times higher than that of normal individuals [105]. Additionally, the presence of acetone in respiratory gases is associated with diabetes [106]. Fiber-based gas sensors can be easily incorporated into masks and facepieces for real-time digital monitoring of biomarkers in the body and hazardous gases outside.

To detect ammonia, Veeralingam et al. designed a self-powered breath ammonia sensor using the TENG, with polypropylene and nylon fabric serving as the friction layers of the generator [107]. Ti-functionalized MoS_2_ was grown on the nylon fiber substrate, and the ammonia molecules in the environment tend to provide the lone electron to MoS_2_, resulting in a decrease in the resistance of the sensor. As an active catalyst, Ti can promote the diffusion and transfer processes of the electrons carried by ammonia to improve the selectivity of the sensor for ammonia. As shown in Figure 8b, an N95 mask embedded with the nanogenerator exhibits a specific sensitivity of 0.512 ppb^−1^ for ammonia with a detection range of 200 ppb to 2600 ppb. The presence of breath acetone (BrAC) indicates diabetes or a lack of carbohydrate intake. It can be seen from Figure 8c that a wireless mask device based on MXene was worn on the volunteer’s face for the detection of acetone gas during respiration [108]. Short peptides and MXene@TiO_2_ were sprayed onto gold electrodes to prepare the acetone sensor, where TiO_2_ nanoparticles were used to enhance the sensitivity of the sensor to acetone and short peptides were used to improve its selectivity. The acetone sensing mechanism is attributed to the interlayer expansion effect of MXene nanosheets. When the sensor is exposed to acetone, the adsorption of gas molecules acting as an intercalating agent increases the interlayer distance of MXene and suppresses interlayer conduction, leading to an increase in the sensor resistance (Figure 8d). When the body lacks carbohydrates for energy, acetone is produced through fat metabolism, so it is possible to respond to lipid metabolism in the body by monitoring acetone in respiratory gases. In Figure 8e, the acetone smart sensor mask measures the dynamic acetone content in the respiration of volunteers under different dietary patterns (balanced diet, ketogenic diet, and carbohydrate diet), demonstrating the potential application of wearable acetone sensors in daily diet monitoring and digital health management.

The relative humidity (RH) of the respiratory system is an important health parameter. The fiber-based humidity sensors can be integrated into masks or clothing to easily determine the level of dehydration in the human body by monitoring RH levels in breathing gas during exercise, which can be useful as a reminder to hydrate instantly [111]. Choosing the right fiber material substrate can speed up the response time to relative humidity, so a resistive humidity sensor has been developed based on Coolmax fibers functionalized with GO [109]. The GO provides abundant oxygen-containing functional groups, which can adsorb water molecules in a humid environment. When the ambient RH is low, water molecules are bound to the GO surface through strong hydrogen bonding and cannot move freely. As the RH increases, more water molecules are bound to the GO surface, forming a water-molecule layer. This process accelerates the conversion of H_2_O to H_3_O, and the free movement of protons during the conversion leads to a decrease in the resistance of the sensor. Because of the presence of grooves and indentations on the surface of Coolmax fibers, the effectiveness of water transport is enhanced, allowing the sensor to reach equilibrium quickly (within 0.15 s). This is beneficial for achieving a fast response in RH detection. Additionally, Figure 8f shows a wearable fabric keyboard using finger humidity as a stimulus to transmit signals. This device provides a new approach to addressing unintentional errors caused by inevitable accidental presses.

Moist-electric nanogenerators can convert humidity variations into electrical energy output without the requirement of external energy input, making them suitable for self-powered moist-electric humidity sensors (MEHS) [112,113]. Tai et al. prepared the MEHS by using MXene/NbC/sodium alginate composite films via the electrospinning method [110]. These films exhibit increasing output voltage (0–0.53 V) with the increase in humidity, high linearity (R^2^ = 0.99), and relatively fast response/recovery time (increasing from 25.0–66.9 s with film thickness). When the MEHS is exposed to a humid environment, water molecules diffuse and ionize along the nanochannels constructed by the network structure. Due to the directional movement of charges, a potential difference is created between the two electrodes, resulting in an output voltage signal. As shown in Figure 8g, the array of MEHSs can charge a capacitor to drive the LED bulb. The brightness of the light bulb visually indicates the changes in relative humidity, eliminating the need for external power sources or additional signal transmission devices.

Applying fiber-based biochemical sensors to monitor individual biomarkers has shown promising potential. However, certain challenges need to be addressed for their widespread implementation. These include enhancing the sensors’ sensitivity and selectivity to accurately detect target analytes while minimizing interference from other substances. Sensors often also have conductive materials, so insensitivity to mechanical stimuli is also something to consider. These materials may emit electrical signals in response to external strain and pressure stimuli, thus affecting the examination of biomarkers. Additionally, ensuring the long-term stability and durability of the sensors in contact with biological fluids remains crucial. Moreover, the scalability and commercialization of these sensors require further investigation, considering cost-effectiveness, large-scale production feasibility, and market demand.

## 4. Conclusions and Perspectives

In summary, the progress of fiber-based wearable sensors for personal digital health was comprehensively investigated and discussed. In the monitoring of biophysical signals, the classification based on sensing various mechanisms (i.e., battery-powered resistive and capacitive sensors and self-powered piezoelectric, triboelectric, and magnetoelastic sensors) of fiber-based sensors was provided. In addition, fiber-based sensors for biological temperature monitoring based on resistive and thermoelectric effects are also described. Subsequently, fiber-based biochemical sensors were introduced according to the different objects, including bodily fluid monitoring (sweat, saliva, and urine) and respiratory gas monitoring. Finally, the tough challenges and future remarks of fiber-based wearable sensors for personal digital health were evaluated and summarized.

Although there have been many studies devoted to improving the performance of fiber-based sensors, there are still some barriers to improvement in their sensitivity, especially in the monitoring of biological temperature and biochemistry. Additionally, the changes in an individual’s temperature and biomarker concentrations are usually subtle and difficult to perceive in poor health conditions. Enhancing the sensitivity of fiber-based sensors to collect accurate personal digital health signals is of significance in practical applications. In addition, great linearity, sensing range, and durability are also important factors for fiber-based wearable sensors to implement long-term and effective motion monitoring and health management. It requires further exploration of materials, structures, and sensing principles. Currently, the size of fiber-based wearable sensors is often limited to yarn or small pieces of fabric. Further integration between sensors and daily clothing may have some impact on the performance of the sensors. This restriction may be attributed to expensive manufacturing costs and intricate manufacturing procedures. Therefore, fabricating them in low-cost mass production is of great significance for long-term detection. Moreover, the monitoring of complex human joints, such as the ankle and hip, is often overlooked in the application of fiber-based sensors. These important joints have more than one degree of freedom and produce complex and variable movements. This inspires the possibility of arraying fiber-based sensors in the future and integrating the data through signal processing techniques to achieve accurate identification of joint movements.

Textiles have natural moisture-wicking and sweat-evaporating properties, which are beneficial for the comfort of fiber-based wearable sensors. However, this property is a potential disadvantage in the monitoring of biofluids. Since some of the biofluids are not excreted in high amounts (e.g., sweat is only released in large quantities during intense activity), it will become more difficult to obtain accurate and continuous monitoring if a significant amount of biofluid evaporates into the environment in a short time. Therefore, for fiber-based biofluid sensors, it is necessary to reduce response time, lower detection limits, and improve sensitivity. To avoid corrosion from biofluids, fiber-based wearable sensors should also possess washability or self-cleaning abilities to ensure stability and durability in long-term use. Encapsulation is always used to prevent biofluid corrosion and reduce the damage caused by external stimuli. Note that the fiber-based biochemical sensors need to directly contact the skin or directly sense exhaled gases, and the presence of an encapsulation layer will cause the textiles to lose their inherent softness and breathability. The encapsulation layer also hinders molecular diffusion, reducing the sensitivity and response speed of the sensor. Future efforts should be directed towards the development of durable, self-cleaning fiber-based sensors without encapsulation to meet the requirements of real application scenarios.

Many battery-powered fiber-based wearable sensors require external power sources for body monitoring, adding equipment cost and extra burden to wearable devices. In the future, the integration of fiber-based wearable sensors with energy storage devices or flexible batteries will enable real-time monitoring of human motions and movements without external power, facilitating medical diagnosis and treatment in personal digital health. Self-powered sensors such as piezoelectric sensors, triboelectric nanogenerators, magnetoelastic generators, and thermoelectric generators are capable of perceiving external changes (such as pressure, friction, temperature, and humidity variations) and converting them into voltage signals. However, these devices still face a dilemma with monitoring static signals such as stationary body posture, constant temperature, or humidity. In response to this issue, it is important to carefully consider the sensor’s potential application scenarios and choose the appropriate sensing technology for the different biosignals of the human body.

In conclusion, fiber-based wearable sensors have demonstrated a significant potential for widespread application in the next generation of wearable personal digital health systems. Developing fiber-based sensors with high-performance and clever designs to achieve multimodal digital health monitoring. By combining flexible fiber display technology and dynamic interaction technology, comprehensive monitoring of individual physical and chemical health signals can be realized throughout the body. This will truly bring fiber-based wearable sensors from the laboratory to human daily life, greatly expanding their application scope and promoting personal and societal health advancement.

## Data Availability

Not applicable.
